# *Tr*-milRNA1 Contributes to Lignocellulase Secretion under Heat Stress by Regulating the Lectin-Type Cargo Receptor Gene *Trvip36* in *Trichoderma guizhouence* NJAU 4742

**DOI:** 10.3390/jof7120997

**Published:** 2021-11-23

**Authors:** Tuo Li, Jinding Liu, Qin Wang, Yang Liu, Ting Li, Dongyang Liu, Qirong Shen

**Affiliations:** 1 Jiangsu Provincial Key Lab of Solid Organic Waste Utilization, Jiangsu Collaborative Innovation Center of Solid Organic Wastes, Educational Ministry Engineering Center of Resource-Saving Fertilizers, Nanjing Agricultural University, Nanjing 210095, China; 2018203039@njau.edu.cn (T.L.); 2020103111@stu.njau.edu.cn (Q.W.); 2021203046@stu.njau.edu.cn (Y.L.); 2020103119@stu.njau.edu.cn (T.L.); shenqirong@njau.edu.cn (Q.S.); 2 College of Information Management, Nanjing Agricultural University, Nanjing 210095, China; liujd@njau.edu.cn

**Keywords:** lignocellulase secretion, *Trichoderma guizhouence* NJAU 4742, heat stress, milRNA, posttranscriptional regulation

## Abstract

Background: MicroRNA plays an important role in multifarious biological processes by regulating their corresponding target genes. However, the biological function and regulatory mechanism of fungal microRNA-like RNAs (milRNAs) remain poorly understood. Methods: In this study, combined with deep sequencing and bioinformatics analysis, milRNAs and their targets from *Trichoderma guizhouence* NJAU 4742 were isolated and identified under solid-state fermentation (SSF) by using rice straw as the sole carbon source at 28 °C and 37 °C, respectively. Results: A critical milRNA, TGA1_S04_31828 (*Tr*-milRNA1), was highly expressed under heat stress (37 °C) and adaptively regulated lignocellulase secretion. Overexpression of *Tr*-milRNA1 (OE-*Tr*-milRNA1) did not affect vegetative growth, but significantly increased lignocellulose utilization under heat stress. Based on the bioinformatics analysis and qPCR validation, a target of *Tr*-milRNA1 was identified as *Trvip*36, a lectin-type cargo receptor. The expression of *Tr*-milRNA1 and *Trvip36* showed a divergent trend under SSF when the temperature was increased from 28 °C to 37 °C. In addition, the expression of *Trvip*36 was suppressed significantly in *Tr*-milRNA1 overexpression strain (OE-*Tr*-milRNA1). Compared with the wild type, deletion of *Trvip*36 (Δ*Trvip*36) significantly improved the secretion of lignocellulases by reducing the retention of lignocellulases in the ER under heat stress. Conclusions: *Tr*-milRNA1 from NJAU 4742 improved lignocellulose utilization under heat stress by regulating the expression of the corresponding target gene *Trvip*36. These findings might open avenues for exploring the mechanism of lignocellulase secretion in filamentous fungi.

## 1. Introduction

Small RNA (sRNA)-induced RNA interference (RNAi) is a broad biological process that can lead to sequence-specific degradation or translational repression of target mRNAs [1,2]. The common feature of RNAi pathways is that sRNAs bind to Argonaute (AGO) proteins and guide the RNA-induced silencing complex (RISC) to RNAs with complementary sequences [3,4]. RNAi is highly conserved in eukaryotes, including the majority of the fungal kingdom [5]. Fungal RNA interference was first discovered in *Neurospora crassa*. Introducing fragments of *albino-1* (*al-1*) or *albino-3* (*al-3*), which are required for carotenoid biosynthesis, reduced *al-1*, or *al-3* mRNA levels and resulted in an albino phenotype [6]. With the expansion of fungal genome information, RNAi pathway components have been found in most fungal species. Studies on *Schizosaccharomyces pombe* and other fungi have revealed various small RNA biogenesis pathways, suggesting that RNAi related pathways are utilized in all cellular processes to adapt to complex external environments [7].

sRNAs, defined by their length of 18–24 nucleotides, play significant roles in growth and development processes, pathogenicity and stress response [2,8,9]. Usually, miRNAs are a class of interior sRNAs found in eukaryotes, that function in many processes, such as development, biotic and abiotic reactions, and defense [10]. Previously, miRNA-like RNAs (milRNAs), with similar characteristics of miRNAs in animals and plants, were recognized and corroborated to be generated through at least four diverse pathways in *N. crassa* [11]. Interestingly, at least four distinct mechanisms have been discovered to produce milRNAs. Dicer-independent small interfering RNAs (disiRNAs) with a size of 21 or 22 nt were also identified in *Neurospora* [11]. Afterward, a large number of milRNAs were identified in fungi. Fifteen milRNAs could regulate mycelium growth and conidiogenesis processes in *Metarhizium anisopliae* [12], and 27 sRNAs were found to have miRNA-like precursor structures in *Botrytis cinerea* [13]. In addition, the presence and differential expression of *Trichoderma reesei* milRNAs when *T. reesei* was cultivated in basal medium supplemented with 3% Avicel (microcrystalline cellulose powder) or 2% glucose, implied that milRNA might function in *T. reesei* growth and cellulase induction [14]. Nevertheless, analogous studies have mainly focused on bioinformatics predictions, but the biogenesis and biological function of milRNAs are rarely investigated.

*Trichoderma guizhouense* NJAU 4742, a saprophytic filamentous fungi isolated from mature compost, has recently attracted wide attention due to its potential to promote plant growth [14,15]. Similar to other heterotrophs, NJAU 4742 usually relies on particular host organisms or substrates for their nutrition. During the colonization process in different habitats including soils and plant roots, NJAU 4742 always feeds on dead fungi or efficiently degrades different plant debris to obtain enough energy resources [16]. Thus, the synthesis and secretion of lignocellulases by NJAU 4742 is one of the critical parameters during the colonization process. Nevertheless, various factors affect its biological functions, especially when the topsoil temperature is too high. Our previous results showed that the secretion of lignocellulases by NJAU 4742 was significantly suppressed at 37 °C (unpublished results). Here, deep sequencing and molecular assays were used to reveal the mechanism of lignocellulase secretion in NJAU 4742 under posttranscriptional regulation mediated by *Tr*-milRNAs. The preliminary analysis of the results indicated that TGA1_S04_31828 (*Tr*-milRNA1) adaptively regulates a lectin-type cargo receptor (*Trvip36*), which might be related to the lignocellulase secretion under heat stress. VIP36 is an intracellular lectin cycling between the endoplasmic reticulum (ER) and the Golgi apparatus, and it is deemed to serve as a cargo receptor in the transport and classification of glycoproteins [17]. VIP36 has also been shown to recycle human α1-antitrypsin from the Golgi compartment back to the ER, and silencing VIP36 improved α1-antitrypsin production. This fact demonstrates that this cargo receptor has a protein retention function [18]. In *Aspergillus oryzae*, deletion of VIP36 improved heterologous protein secretion, and the results of ER-enriched cellular fractions revealed that VIP36 was involved in the retention of heterologous proteins in the ER [19]. As VIP36 could form a stable complex with the molecular chaperone BiP, it appeared to be involved in the quality control of secretory proteins [17]. Therefore, lectin-type cargo receptors might alter the secretion of cellulases, which would be potential suitable models for studying the relationship between cargo receptors and secretory proteins in filamentous fungi.

The objective of this study was to uncover the role of *Tr*-milRNA in lignocellulose utilization of NJAU4742 under heat stress. Further studies investigated the *Tr*-milRNA1 regulation mechanism and the involvement of lectin-type cargo receptors in the intracellular transfer of cellulases in NJAU 4742 under heat stress, which aids readers to have a deeper understanding of lignocellulose biodegradation by filamentous fungi.

## 2. Materials and Methods

### 2.1. Strains and Culture Conditions

*T. guizhouense* NJAU 4742 isolated from a compost sample was maintained in our laboratory, and its genome sequence was deposited in the NCBI database (Accession No. LVVK00000000.1). Mandels’ salt solution without organic components (1.4 g L^−1^ (NH_4_)_2_SO_4_, 2.0 g L^−1^ KH_2_PO_4_, 0.3 g L^−1^ CaCl_2_, 0.3 g L^−1^ MgSO_4_, 5 mg L^−1^ FeSO_4_·7H_2_O, 20 mg L^−1^ CoCl_2_, 1.6 mg L^−1^ MnSO_4_ and 1.4 mg L^−1^ ZnSO_4_) supplemented with 2% (*w*/*v*) rice straw was used for lignocellulase production under SSF [20]. Spores were obtained from incubated PDA plates followed by filtration with four layers of sterilized gauze, and the spore suspension was adjusted to 1.0 × 10^7^ spores·mL^−1^ by quantifying spores on a hemocytometer.

### 2.2. Small RNA cDNA Library Construction and High-Throughput Sequencing

Mycelial samples were harvested after 72 h by using rice straw as the sole carbon source at 28 °C (T28) and 37 °C (T37). Total RNA was extracted using the RNeasy^®^ Plant Mini Kit (Qiagen, Hilden, Germany) and handled with DNase I (TaKaRa) by following the manufacturer’s instructions. RNA concentration and purity were evaluated in an Agilent 2100 Bioanalyzer (Agilent, Santa Clara, CA, USA) to check RNA integrity. Sequencing libraries were generated by employing NEBNext^®^ Multiplex Small RNA Library Prep Set for Illumina^®^ (New England BioLabs Inc., Beverly, MA, USA) according to the manufacturer’s instructions. The clustering of each index-coded sample was carried out on a cBot Cluster Generation System using TruSeq SR Cluster Kit v3-cBot-HS (Illumina, San Diego, CA, USA) according to the manufacturer’s instructions. Finally, libraries were sequenced through an Illumina HiSeq 2500 platform.

### 2.3. Tr-milRNAs Sequence Analysis and Target Prediction

The original reads from sequencing data were filtered by removing poor quality reads, adaptor contamination reads and reads longer than 30 nt or shorter than 18 nt. The standard-compliant reads of small RNAs were aligned to the reference NJAU 4742 genome (https://bioinfo.njau.edu.cn/tgn4742/, accessed on 20 November 2021). The alignment analysis was performed on the CLC Genomics Workbench 12. The sequences that corresponded to known miRNAs were defined by matching to the miRNA database (miRBase 22.0) [21]. The unannotated sRNA sequences were aligned to the reference NJAU 4742 genome to identify precursor sequences for novel miRNAs. Novel miRNAs were predicted by miRDeep2 with a stem-loop structure [22]. The R package DEGseq software was used to analyze the differentially expressed miRNAs. TargetFinder and psRNATarget were used to predict the candidate target genes of *Tr*-milRNAs [23,24].

### 2.4. Relative Expression of Tr-milRNA1 and the Corresponding Target Genes

Samples of different treatments were collected at 0, 24, 36, 48, 60 and 72 h post inoculation (hpi). Total RNA was extracted using the RNeasy^®^ Plant Mini Kit (Qiagen, Germany) according to the manufacturer’s instructions. Expression of *Tr*-milRNA1 was determined by stem-loop qRT–PCR as previously described [25]. First strand cDNA was synthesized by miRNA First Strand cDNA Synthesis (Vazyme Biotech, Nanjing, China) with the stem-loop RT primer based on the manufacturer’s instructions. PCR detection was performed by a *Tr*-milRNA1-specific forward primer and a universal reverse primer. The NJAU 4742 18S rRNA biogenesis gene (18S) was used as a control. qRT–PCR was performed using the miRNA Universal SYBR qPCR Master Mix (Vazyme Biotech, Nanjing, China) according to the manufacturer’s instructions. For the determination of transcript levels of the corresponding target genes, cDNA synthesis was completed by the PrimeScript RT Reagent Kit (RR036A, Takara, Dalian, China) according to the manufacturer’s instructions. qRT-PCR was performed using SYBR Premix Ex Taq II (RR820A, Takara, Dalian, China) and the CFX connect^TM^ Real-Time system (Bio–Rad, Hercules, CA, USA). Transcription levels of the target genes were normalized by the 2^−ΔCt^ method, and translation elongation Factor 1 alpha (*Tef*) was used as the housekeeping gene. All primers used in this study are listed in Table S1.

### 2.5. Deletion and Overexpression of Fungal Small RNAs

For targeted deletion of *Tr*-milRNA1, the 5′ and 3′ flanking regions of *Tr*-milRNA1 were amplified by PCR from NJAU 4742 genomic DNA. *HygB* was used as a resistance gene and the fragment was amplified by PCR from plasmid pcDNA1 (Vienna University of Technology). The three fragments were fused by CloneAmp HiFi PCR Premix (Takara, Japan) according to the manufacturer’s instructions. The *Tr*-milRNA1 deletion mutant was generated via a gene replacement strategy using the polyethylene glycol (PEG)-mediated protoplast transformation procedure as described in Zhang et al. [26].

To overexpress *Tr*-milRNA1 in NJAU 4742, the 400 bp fragment surrounding primary *Tr*-milRNA1 (a fragment of approximately 200 bp upstream and downstream of primary *Tr*-milRNA1) was amplified by PCR from NJAU 4742 genomic DNA and then introduced into plasmid pcDNA1 by the ClonExpress-II One Step Cloning Kit (Vazyme Biotech, Nanjing, China). In pcDNA1: *Tr*-milRNA1 precursor construct was expressed under the control of the cDNA1 promoter (Figure S1b). Mutated *Tr*-milRNA1 (OE-Mut-*Tr*-milRNA1) expression construct was obtained by a Fast Site-Directed Mutagenesis Kit (Tiangen) according to the manufacturer’s instructions. The sequence of constructs was confirmed by sequencing (Tongyong Biological Technology, Chuzhou, China). The vectors of OE-*Tr*-milRNA1, OE-Mut-*Tr*-milRNA1 and empty vector were separately transformed into NJAU 4742 by using the polyethylene glycol (PEG)-mediated protoplast transformation procedure [26]. The relative expression level of *Tr*-milRNA1 between wt, mutants OE-*Tr*-milRNA1, OE-Mut-*Tr*-milRNA1 and empty vector transformant (EV) was measured following the above method (relative expression of *Tr*-milRNA1 at different sampling times). A diagram shows the strategy of *Tr*-milRNA editing and mutant detection used in this study (Figure S1a,b,d,f,g), and the primers are given in Table S1.

### 2.6. Generation of the Target Gene Mutants

To obtain single knockout mutant homologous recombination fragments of *Trvip36*, the 5′ and 3′ flanking regions of the gene open-reading frame (ORF) were amplified by PCR from NJAU 4742 genomic DNA, and the two fragments were ligated with *Hygb* through CloneAmp HiFi PCR Premix (Clontech) according to the manufacturer’s instructions. To generate a *Trvip36* overexpression mutant homologous recombination fragment, three fragments including the 5′ flanking regions of the gene ORF, the promoter fragment and the ORF of the *Trvip36* gene fragment were amplified by PCR from NJAU 4742 genomic DNA and then fused with *Hygb* by using CloneAmp HiFi PCR Premix (Clontech). *Trvip36* deletion and overexpression mutants were generated by using homologous recombination and a polyethylene glycol (PEG)-mediated protoplast transformation system [26]. A diagram shows the strategy of gene editing and mutant detection used in this study (Figure S1a,c,e), and the primers are given in Table S1.

### 2.7. Growth and Enzyme Activity Assays of NJAU 4742

Equally harvested biomass samples of different treatments were transferred to the medium with rice straw as the sole carbon source and incubated at 37 °C to determine the growth rate. For enzyme activity assays, 1 mL of fresh spore suspension (1.0 × 10^7^ spores·mL^−1^) of different strains was inoculated for SSF. All samples of different treatments were collected on the 4th day, and three biological replicates were collected at each sampling point. Filter paper activity (FPA) and endoglucanase activity (EG) were measured according to the method described by Xue et al. [27] with filter paper (Whatman NO.1) and CMC-Na (Sigma, St. Louis, MO, USA) as the substrates. Xylanase activity (XYL) was assayed with oat spelts xylan (Sigma, St. Louis, MO, USA) as the substrate [28]. The reaction system was executed in 0.1 M acetate buffer (pH 4.8) at 50 °C for 10 min, after which the DNS method was used to measure the released reducing sugars. The cellobiohydrolase activity (CBH) was determined in 0.1 M acetate buffer at 50 °C for 30 min with pNPC (Sigma, St. Louis, MO, USA) as the substrate according to Liu et al. [29]. One enzyme activity unit was defined as the amount of enzyme required to liberate 1 μmol glucose or pNP per minute under the assayed conditions.

### 2.8. Confocal Imaging of EGL-GFP and CBH-GFP in the ER and Golgi Apparatus between wt and ΔTrvip36

The DNA fractions of the representative lignocellulase genes including endoglucanase (*egl*, A1A110863.1) and cellobiohydrolase (*cbh*, A1A102028.1) were amplified by DNA of NJAU 4742, and the eGFP fragment was amplified by using plasmid pEGFP-N1 (Clontech, Mountain View, CA, USA). The DNA fractions of different cellulase genes were ligated with the GFP fragment by the overlapping-PCR technique based on the instructions of CloneAmp HiFi PCR Premix (Clontech, Mountain View, CA, USA). Mutants of endoglucanase-GFP (*egl-*GFP) and cellobiohydrolase-GFP *(cbh*-GFP) were obtained through homologous recombination based on wt and Δ*Trvip36* (Figures S1h and S2). Subsequently, these mutants labeled with GFP in situ were cultured in medium with rice straw as the sole carbon source for three days, and the freshly cultured mycelium was incubated with ER-Tracker™ Red (Thermo Fisher Scientific, Cat. M7512, Ex/Em = 587 nm/615 nm) or the BODIPY^TM^ TR Ceramide (Thermo Fisher Scientific, D7540, Ex/Em = 589 nm/616 nm) at a concentration of 100 nM at room temperature for 5 min in darkness. Subsequently, the cellulase-GFP and ER or Golgi apparatus stained hyphae were imaged under a confocal fluorescence microscope (TCS SP8, Leica, Germany), and the fluorescence intensity and colocalization analysis were performed by plot profile in ImageJ software according to Zhao et al. [30]. All fluorescence intensity values of EGL-GFP and CBH-GFP in the ER and Golgi apparatus were counted when the fluorescence intensity value of ER-Tracker or Golgi-Tracker was valid (higher than 0). Data were completed as at least three independent biological replicates. Statistical data were expressed as means ± standard errors (SE) from all valid pixels.

## 3. Results

### 3.1. Identification and Quantification of milRNAs in NJAU 4742

**Figure 1:**
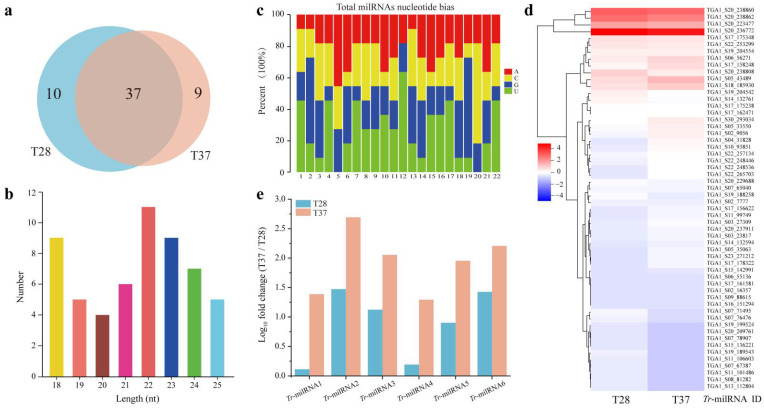
*Tr-*milRNAs are differentially expressed under SSF using rice straw as the sole carbon source at 28 °C (T28) and 37 °C (T37). (**a**) Different quantities of *Tr-*milRNAs were isolated from T28 (blue) and T37 (orange). Ten *Tr*-milRNAs and nine *Tr*-milRNAs are expressed specifically at T28 or T37, respectively. Thirty-seven *Tr*-milRNAs are coexpressed in T28 and T37; (**b**) length distribution of *Tr*-milRNAs; (**c**) nucleotide bias of *Tr*-milRNAs; (**d**) Log_10_ (TPM) normalized values of all *Tr*-milRNAs were used to generate the heatmap. Red and blue colors indicate relatively high and low *Tr*-milRNA expression, respectively, and white equals median abundance; (**e**) normalized read numbers of *Tr*-milRNAs with transcripts per million (TPM) values higher than 10 in T28 or T37.

In order to determine whether milRNAs are involved in the regulation of cellulase secretion in NJAU 4742 under heat stress, two small RNA libraries were generated from NJAU 4742, grown using rice straw rice straw as the sole carbon source at 28 °C (T28) and 37 °C (T37), respectively. In total, 56 putative milRNAs were identified in NJAU 4742, and 47 and 46 *Tr*-milRNAs were identified at T28 and T37, respectively (Table 1 and Figure 1a). *Tr*-milRNAs were enriched at 22 nt and seemed to possess a strong preference for uracil (Figure 1b,c). Interestingly, 10 and 9 *Tr*-milRNAs were specifically expressed at T28 or T37, respectively. Thirty-seven *Tr*-milRNAs were coexpressed in T28 and T37.

After normalization, 20 *Tr*-milRNAs with transcripts per million (TPM) values higher than 10 were identified. TGA1_S20_236772 and TGA1_S20_238808 exhibited a high abundance at 28 °C. In contrast, TGA1_S04_31828 (*Tr*-milRNA1), TGA1_S02_9056, TGA1_S05_43489 (*Tr*-milRNA2), TGA1_S06_56271 (*Tr*-milRNA3), TGA1_S10_93851 (*Tr*-milRNA4), TGA1_S17_158248 (*Tr*-milRNA5), TGA1_S17_162471, TGA1_S17_175238, TGA1_S17_175348, TGA1_S18_185930 (*Tr*-milRNA6), TGA1_S19_204554, TGA1_S20_223477, TGA1_S20_238860, TGA1_S20_238862, TGA1_S22_248446, TGA1_S22_248536, TGA1_S22_253299, TGA1_S22_257134 and TGA1_S30_293034 showed a high abundance at 37 °C (Table 1 and Figure 1d). At a false discovery rate <0.05 and log_10_ fold change >1 or <−1, six significantly upregulated *Tr*-milRNAs including *Tr*-milRNA1, *Tr*-milRNA2, *Tr*-milRNA3, *Tr*-milRNA4, *Tr*-milRNA5 and *Tr*-milRNA6 were selected (Figure 1e). This result indicated that at least several *Tr*-milRNAs might be involved in the regulation of lignocellulose utilization at different temperatures.

### 3.2. Identification of Candidate Target Genes Related to Tr-milRNAs of NJAU 4742

To investigate target genes of putative *Tr*-milRNAs, two rigorous target prediction software programs (TargetFinder and psRNATarget) were used to predict potential targets in the coding sequences, 3′UTR and 5′UTR of the NJAU 4742 genome. Gene ontology (GO) enrichment analysis indicated that candidate target genes were predictably involved in metabolic process, cellular process, single-organism process, catalytic activity, cell and membrane binding (Figure S3a). *Tr*-milRNA target genes also seem to be enriched through KEGG pathways including carbohydrate metabolism, catabolism, transport and translation (Figure S3b). To further ascertain whether the *Tr*-milRNA target genes were involved in the utilization of lignocellulose at different temperatures, putative target genes predictably involved in the secretion, synthesis and transportation of lignocellulases were selected. These findings might reveal that the differences in lignocellulose utilization at different temperatures are precisely regulated by *Tr*-milRNAs and their targets.

### 3.3. Tr-milRNA1 Exhibits a Critical Role in Lignocellulose Utilization at Different Temperatures

**Figure 2:**
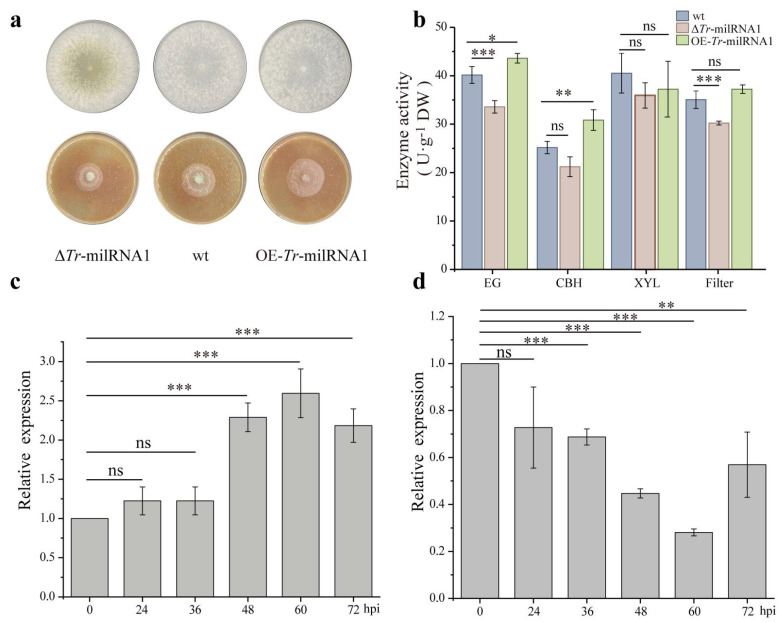
Overexpression of *Tr*-milRNA1 increased cellulase activities by silencing the target gene *Trvip36*. (**a**) Biomass comparison of wt and mutants on PDA at 28 °C (top row) and rice straw medium at 37 °C (bottom row); (**b**) the hydrolase activities, including FPA, EG, CBH and XYL of wt and mutants by using rice straw as the sole carbon source under heat stress; (**c**) relative expression levels of *Tr*-milRNA1 from 0 to 72 hpi at 37 °C. Relative expression levels of *Tr*-milRNA1 were normalized to gene 18S rRNA and calibrated to the levels of wt at 0 hpi (set as 1.0). (**d**) relative expression levels of target *Trvip36* from 0 to 72 hpi at 37 °C. Relative expression levels of *Trvip36* were normalized to the *Tef* gene and calibrated to the levels of wt at 0 hpi (set as 1). Data were calculated from three biological replicates, and error bars represent ± SDs. * *p* < 0.05, ** *p* < 0.01, *** *p* < 0.001. A *p* value < 0.05 was regarded as statistically significant and ns refers to no significance.

To explore the possible roles of *Tr*-milRNAs in lignocellulose utilization at different temperatures, overexpression transformants of *Tr*-milRNAs that were significantly upregulated at 37 °C were generated to screen their function under heat stress. The results showed that *Tr*-milRNA1 overexpression mutant (OE-*Tr*-milRNA1) could significantly increase lignocellulose utilization and also exhibited faster growth rate of mycelium using rice straw as the sole carbon source under heat stress than wt. Additionally, the pri-*Tr*-milRNA1 (primary transcript of *Tr*-milRNA1) deletion mutant (Δ*Tr*-milRNA1) exhibited normal growth, but showed a slight reduction in lignocellulose utilization capacity (Figure 2a). Various lignocellulosic enzyme activities were systematically assessed to determine the utilization efficiency of lignocellulose for filamentous fungi [31]. Therefore, different lignocellulases, including endoglucanase (EG), cellbiohydrolase (CBH), xylanase (XYL), and filter paper activity (FPA) were detected under SSF in different treatments. Noteworthy, EG and CBH activities increased significantly compared with wt under heat stress (*p* < 0.05). Correspondingly, various lignocellulosic enzyme activities of Δ*Tr*-milRNA1 decreased at different levels compared to wt (Figure 2b). These findings suggested that *Tr*-milRNA1 might be critical in regulating the utilization of lignocellulose at different temperatures.

### 3.4. Expression of Trvip36 Could Be Regulated by Tr-milRNA1

Among the preselected target genes of *Tr*-milRNA1, we found that *Tr*-milRNA1 could base pair with several genes predictably related to lignocellulose utilization, including genes encoding carbohydrate hydrolases, transport proteins, and energy homeostasis related proteins. To determine the interaction between *Tr*-milRNA1 and the candidate target genes, we performed target verification tests. The relative expression of *Tr*-milRNA1 and predicted target genes were determined using stem-loop qRT–PCR and qRT–PCR to determine if the expression levels of *Tr*-milRNA1 and candidate target genes are correlated. During SSF, *Tr*-milRNA1 was upregulated at 36, 48, and 60 hpi and downregulated at 72 hpi (Figure 2c). Interestingly, we found that the transcript levels of the lectin-type cargo receptor gene *Trvip36* were downregulated at 0 to 60 hpi and then upregulated at 72 hpi (Figure 2d). However, the results for the other predicted target genes were illogical and contradictory. Thus, the negatively correlated expression of *Tr*-milRNA1 and *Trvip36* might indicate that *Trvip36* could be target of *Tr*-milRNA1.

### 3.5. Tr-milRNA1 Regulates Target Gene Expression in a Sequence-Specific Manner

**Figure 3:**
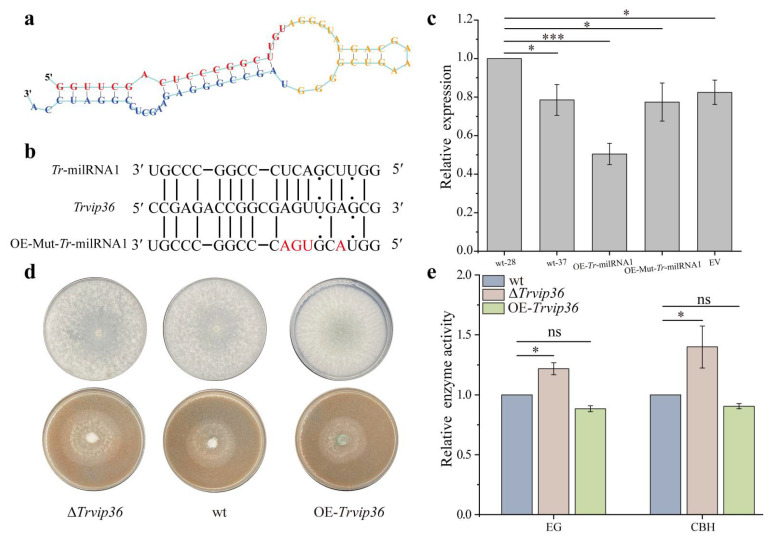
The function of *Tr*-milRNA1 in regulating the target gene *Trvip36*. (**a**) The precursor of *Tr*-milRNA1 could form a hairpin structure. The red line indicates the sequence of mature *Tr*-milRNA1; (**b**) alignment of *Tr*-milRNA1 and mutated *Tr*-milRNA1 (OE-Mut-*Tr*-milRNA1) with target gene (*Trvip36*) at the predicted binding sites; (**c**) relative expression of *Trvip36* in wt at 28 °C, and in wt, OE-*Tr*-milRNA1, OE-Mut-*Tr*-milRNA1 and EV at 37 °C. Relative expression of *Trvip36* was normalized to the *Tef* gene and calibrated to the levels of wt at 28 °C (set as 1.0). Data were calculated from three biological replicates, and error bars represent ± SDs. * *p* < 0.05, *** *p* < 0.001. A *p* value < 0.05 was regarded as statistically significant and ns refers to no significance; (**d**) growth of wt and different mutants on PDA at 28 °C (top row) and rice straw medium at 37 °C (bottom row), respectively; (**e**) relative activities of EG and CBH between wt, Δ*Trvip36* and OE-*Trvip36* by using rice straw as the sole carbon source under heat stress.

*Tr*-milRNA1 had a 64 bp putative precursor, which might form a typical hairpin structure (Figure 3a). To confirm the regulatory mechanism of *Tr*-milRNA1, a mutated *Tr*-milRNA1 overexpression transformant (OE-Mut-*Tr*-milRNA1) was generated by site-specific mutagenesis (Figure 3b). Subsequently, the transcript level of *Trvip36* was quantified in wt, OE-*Tr*-milRNA1, OE-Mut-*Tr*-milRNA1, and EV at 60 hpi under SSF. Interestingly, the expression of *Trvip36* was significantly suppressed in OE-*Tr*-milRNA1, but not in OE-Mut-*Tr*-milRNA1 or EV (Figure 3c). These results suggested that *Tr*-milRNA1 might regulate the expression of *Trvip36* in a sequence-specific manner.

### 3.6. Deletion of Trvip36 Improved the Secretion of Lignocellulases of NJAU 4742 under Heat Stress

Deletion (Δ*Trvip36*) and overexpression (OE-*Trvip36*) strains of *Trvip36* were generated to determine the function of lignocellulose utilization under heat stress. Compared to wt, both mutants showed no difference in vegetative growth (Figure 3d). Nevertheless, Δ*Trvip36* and OE-*Trvip36* exhibited opposite growth trends, and Δ*Trvip36* showed better growth rate relative to wt by using rice straw as the sole carbon source under heat stress (Figure 3d). Previous results showed that the EG and CBH activities increased significantly in OE-*Tr*-milRNA1. Thus, these two enzyme activities were also measured in Δ*Trvip36* and OE-*Trvip36* to determine whether these changes were associated with the expression level of *Trvip36*. In correlation with *Trvip36*, EG and CBH activities increased significantly in Δ*Trvip36* and slightly decreased in OE-*Trvip36* (Figure 3e). These findings suggested that activation of *Trvip36* might suppress lignocellulase secretion, and we concluded that impaired expression of *Trvip36* regulated by *Tr*-milRNA1 contributes to the utilization of lignocellulose under heat stress.

### 3.7. Deletion of Trvip36 Reduces the Retention of Lignocellulases in the ER of NJAU 4742 under Heat Stress

**Figure 4:**
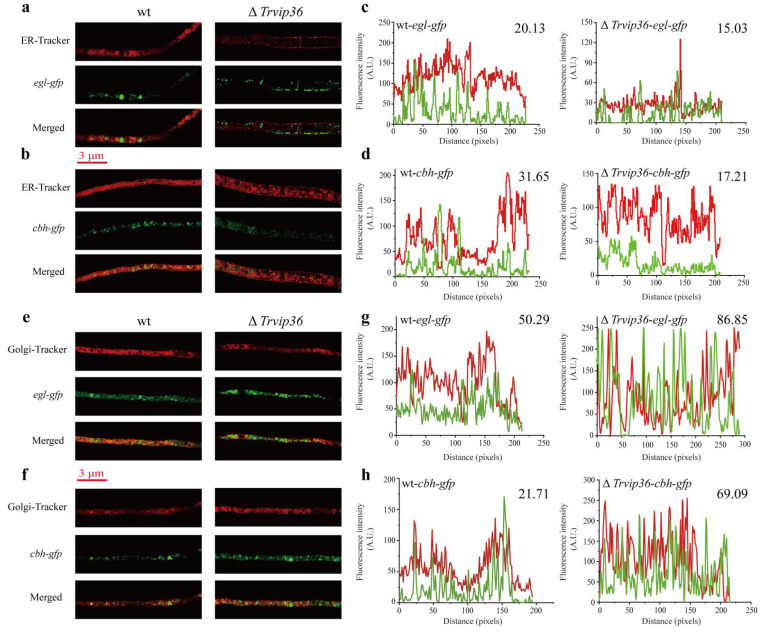
Localization analysis of EGL*-*GFP and CBH-GFP in wt and Δ*Trvip36* genetic backgrounds after staining hyphae with ER-Tracker™ and BODIPY^TM^ TR Ceramide, as markers of ER and Golgi compartments. (**a**,**b**) Confocal images of hyphae from *egl-*GFP and *cbh*-GFP fusion strains after staining with ER-Tracker^TM^ Red to track endoglucanase and cellobiohydrolase localization in the ER between wt and Δ*Trvip36*. Bar = 3 μm; (**c**,**d**) the fluorescence intensity of the two channels (ER-Tracker (red) and GFP (green)) detected in wt and Δ*Trvip36*; (**e**,**f**) confocal images of hyphae from the *egl-*GFP and *cbh*-GFP fusion strains after staining with BODIPY^TM^ TR ceramide to track endoglucanase and cellobiohydrolase localization in the Golgi apparatus between wt and Δ*Trvip36*. Bar = 3 μm; (**g**,**h**) the fluorescence intensity of the two channels (Golgi-Tracker (red) and GFP (green)) detected in wt and Δ*Trvip36*.

In mammalian cells, VIP36 dynamically localized to the cis-Golgi and ER-Golgi intermediate compartment, and modification of its N-linked carbohydrate together with other evidence suggested that VIP36 possessed a role in trafficking through later Golgi compartments [32,33]. VIPL, a VIP36-like protein, is widely expressed in animal cells and mainly localizes to the ER [34]. In filamentous fungi, VIP36 was also found to localize in the ER and Golgi compartments and a higher fraction of VIP36 co-localized with the Golgi marker (Golgi protein *Ao*Grh1 fused to EGFP) than with the ER marker (the ER membrane protein *Ao*ClxA fused to EGFP) [19]. We inferred that they might interfere with the normal intracellular transport of cellulases. Thus, strains expressing *egl*-GFP or *cbh*-GFP fusion proteins were generated in wt and Δ*Trvip*36 genetic backgrounds to track their intracellular localization. Cellulases tagged with eGFP were successfully expressed in hyphae of different mutants. To elucidate the aggregation level of cellulases in the ER and Golgi apparatus, the fluorescence intensities of EGL-GFP and CBH-GFP were detected in the ER and Golgi apparatus after staining with ER-Tracker™ Red and BODIPY^TM^ TR Ceramide, respectively (Figure 4a,b,e,f). EGL-GFP and CBH-GFP intensities were analyzed in each pixel by ImageJ software. The average intensity of GFP fluorescence was calculated to measure the expression levels of EGL-GFP and CBH-GFP in the ER and Golgi apparatus. In wt, the average intensity of EGL-GFP and CBH-GFP was 20.13 ± 1.70 and 31.65 ± 1.32 in the ER, 50.29 ± 1.60 and 21.71 ± 2.03 in the Golgi apparatus, respectively. Compared to wt, Δ*Trvip36* exhibited lower average intensities of EGL-GFP (15.03 ± 0.99) and CBH-GFP (17.21 ± 0.94) fluorescence in the ER (*p* < 0.01). In contrast, higher average fluorescence intensities levels of EGL-GFP (86.85 ± 4.01) and CBH-GFP (69.09 ± 3.18) were distributed in the Golgi apparatus of Δ*Trvip36* (*p* < 0.001) (Figure 4c,d,g,h and Figure S4). These results suggest that VIP36 could interfere with the secretion of EGL and CBH by increasing the ER retention rate, which is consistent with the increased secretion of EGL and CBH in Δ*Trvip36*.

## 4. Discussion

Normally, most soil fungi experience a constantly fluctuating environment. Throughout the evolution process, fungi have developed different modes of reproduction and the ability to adapt to complex environments [35]. Generally, fungi operate in narrower temperature ranges, and a decrease in temperature may cause fungal dormancy, while an increase in temperature would lead to the apoptosis of fungi [36]. In fact, a substantial increase in temperature causes attenuation and eventually leads to the death of many organism [37]. The changes associated with heat stress responded to a set of proteins that promoted the survival of the organism. A major portion of these proteins were termed heat-shock proteins (Hsps). Hsps play a major role in cell cycle progression, replication, transcription and post-translational processes, including protein folding, stability, transportation and degradation, and they were also reported to activate many key signal transducers in fungi [38,39]. Therefore, filamentous fungi could precisely regulate the expression of specific genes to maintain essential biological functions under high temperature environment.

In our previous study, we found that cellulase secretion in strain NJAU 4742 was largely limited under heat stress during the SSF process, which seriously inhibited its ability to obtain carbon source. In addition, through proteomic analysis, it was found that some differentially expressed genes responded to temperature changes to regulate cellulase secretion (unpublished data). In this study, we first reported that *Tr*-milRNA was involved in the regulation of cellulase secretion under heat stress. It is generally accepted that miRNAs from plants are integral components of plant responses to disadvantageous environmental conditions [40,41]. For example, in *Arabidopsis*, miR398 was shown to be quickly induced under heat stress, accompanied by the downregulation of its target genes in response to heat stress [42]. Similarly, miR156 could accelerate the sustained expression of heat stress responsive genes through SPL genes [43]. Here, *Tr*-milRNA1, from filamentous fungi NJAU 4742, was revealed to be related to cellulase secretion under heat stress during SSF by regulating its target gene *Trvip36*.

**Figure 5:**
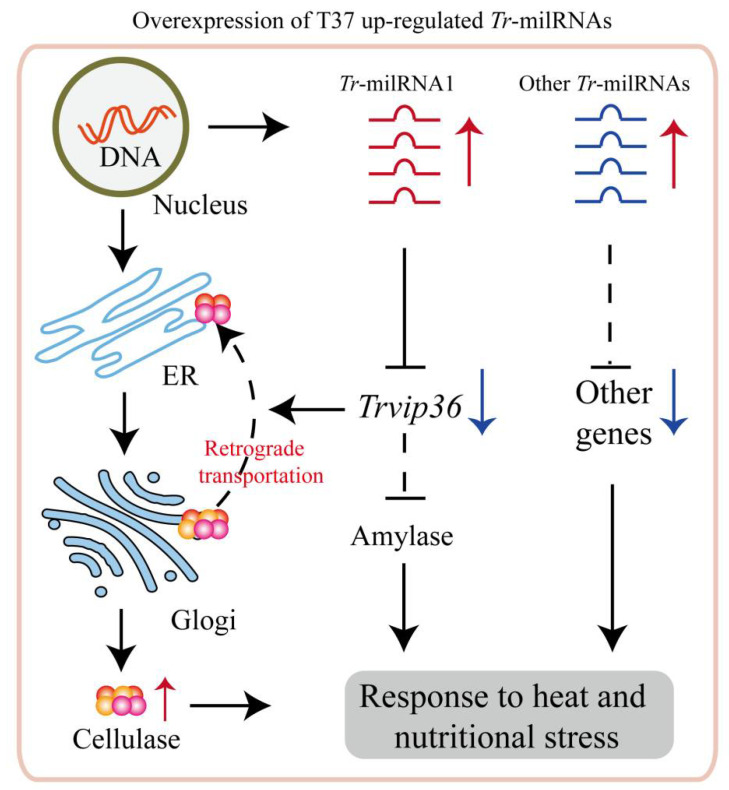
Proposed model for the regulation of NJAU 4742 by *Tr*-milRNAs during the lignocellulose utilization process under heat stress. *Tr*-milRNA1 is involved in regulating cellulase secretion by revoking the expression of *Trvip36* to decrease the cellulase retrograde transportation.

Although numerous milRNAs have been identified in fungi [44,45], no homologous miRNAs were found in NJAU 4742 by mapping the clean reads of *Tr*-milRNA1 to miRBase 22.0. A similar result was discovered in *T. reesei* and *F. oxysporum*, which indicated a large degree of species specificity of fungal milRNAs [46]. More interestingly, there have been few relevant studies on the regulation of cellulase secretion by milRNAs, especially under heat stress. Previously, differential expression of milRNAs in *T. reesei* was discovered when *T. reesei* was cultivated in Avicel medium or glucose medium, which implied that milRNA might be involved in fungal growth and cellulase secretion [14]. In *Ganoderma lucidum*, several milRNA target genes were also identified by high-throughput sequencing and degradome analysis, and were considered glycoside hydrolases involved in the biosynthesis of polysaccharides [47]. Thus, it seemed that milRNAs were related to the regulation of cellulose degradation but there was a lack of in-depth evidence. In plants and animals, miRNAs could play vital regulatory roles by targeting mRNAs for cleavage or translational repression [10], while the regulatory mechanism of milRNAs in fungi is largely unknown. In this study, *Tr*-milRNA1 negatively regulated the gene expression of lectin-type cargo receptor by suppressing the expression of *Trvip36*. There was evidence that *Tr*-milRNA1 regulated target gene expression in a sequence specific mode. During SSF, upregulation of *Tr*-milRNA1 increased the repression effect of *Trvip36*, which promoted cellulase secretion and exhibited better growth rate under heat stress. Deletion mutant of *Trvip36* also showed a significant increase in cellulosic enzyme activities, which might be due to the reduction in cellulase retention in the ER compared to wt. Consistent with this speculation, in *Aspergillus oryzae*, *AoVip*36 was shown to have a protein retention function in the ER and the deletion of *AoVip*36 could increase the secretion of heterologous proteins [19]. In mammals, glycoprotein α1-antitrypsin was determined to be the retention target of VIP36, and it exhibited a significant promotion of intracellular transport upon silencing VIP36 [18]. VIP36 could also form a complex with BiP independently to increase its N-glycan binding activity, and overproduction of a lectin-deficient form of VIP36 could decrease the secretion of clusterin [17]. These results suggested that VIP36 could also negatively interfere with protein transport in an N-glycan-independent manner [48]. In addition, it was reported that VIP36 could be recycled between the Golgi and the ER and carry out the retrograde transport of glycoproteins [49]. Moreover, it is worth noting that heat and nutrient deprivation stress could lead to the generation of protein aggregates in the ER [50]. Avoiding the excessive aggregation of proteins in the ER could alleviate ER stress, which would be conducive to protein synthesis and secretion [51]. Thus, we speculated that deletion of *Trvip36* might also contribute to reducing the retrograde transport of lignocellulases and preventing the aggregation of cellulases in the ER to relieve ER stress thereby increasing the secretion of cellulases by using rice straw as the sole carbon source under heat stress. In addition, it was reported that deletion of VIP36 would impair the secretion of α-amylase [19,52], which might suggest that some unnecessary pathways would be decreased to avoid excessive energy consumption of filamentous fungi under stress (Figure 5). In fact, the main component of rice straw is lignocellulose [53], and producing more lignocellulases is the most important way to obtain energy and maintain vegetative growth, especially under heat stress. However, its internal regulatory mechanism between *Trvip36* and the secretion of α-amylase in NJAU 4742 still needs in-depth and detailed study in the future.

## 5. Conclusions

Overall, this study demonstrated that *Tr*-milRNA1 from NJAU 4742 played a critical role in cellulase secretion by regulating the endogenous target gene *Trvip36*. These results provide important evidence to determine the roles of milRNA and their corresponding target gene during the utilization of lignocellulose in filamentous fungi under heat stress.

## Figures and Tables

**Table 1:** Identification and expression abundance of milRNAs in NJAU 4742 by miRNA deep sequencing.

miRNA_ID	Mirdeep2_Score	RNAfold_Results	TPM (T28)	TPM (T37)	Mature_Sequence
TGA1_S20_236772	2,295,965.4	yes	983,492.79	976,779.71	uuuuugagauacuccgcaacgac
TGA1_S20_238860	31,534.5	yes	11,201.07	16,331.53	uacguaggacuuuaccgugacgu
TGA1_S06_56271	115.5	yes	12.30	112.44	uugccgaguggcagaggacuggcau
TGA1_S17_175348	105	yes	23.71	47.53	uggaaguugaaucgagaagcccu
TGA1_S17_158248	85.8	yes	6.96	89.12	gcaucugauuuccacccuuggguu
TGA1_S19_204554	43.5	yes	10.77	28.55	agcuuuuggcuuuccagaacccgu
TGA1_S19_204542	38	yes	11.73	11.75	uggcuggacggcccagagggccu
TGA1_S30_293034	14.6	yes	2.86	35.64	cgcaggcucgauuguugucu
TGA1_S14_132594	13.2	yes	0.18	4.88	cgccaacggcccucgcccggcuac
TGA1_S19_188258	12.5	yes	2.18	2.91	caucugcauguugucucugg
TGA1_S10_93851	11.9	yes	0.56	18.67	gucuagcacucuacuuuggcau
TGA1_S17_156622	9.3	yes	0.30	6.57	uuuuuacacagauaccaguaggu
TGA1_S20_237911	7.5	yes	0.77	5.62	ccaccaggccgcucaagacua
TGA1_S03_23817	7.1	yes	0.68	5.62	cacugaccugcuccucgcacag
TGA1_S17_161581	7.1	yes	0	1.69	caucuagcuuggacggcagcg
TGA1_S11_99749	6.5	yes	0.30	5.62	ucgucacccuugaggcggaa
TGA1_S02_7777	5.4	yes	0.86	3.66	auugcgaugccuggucagcuacuc
TGA1_S22_248536	5.4	yes	0.38	12.40	ugaggagucugaagauggagaggaa
TGA1_S03_27309	5.3	yes	0.60	7.80	guggacagauuagcugacccgcggg
TGA1_S15_136221	4.5	yes	0.36	0	accaacagcggacauugcuccaa
TGA1_S11_101486	4	yes	0.60	0	ugcuccaaaugagaaucgaagu
TGA1_S11_106603	3.7	yes	0.76	0	ugccugguacauguacgga
TGA1_S07_71495	3.5	yes	1.80	0.85	ugaccaagaacuucgacgucuu
TGA1_S15_142991	3.3	yes	0	2.54	uuuggaccgugucuggaacgcua
TGA1_S17_178322	3.3	yes	0	5.62	ucauauucucagcacuuggaau
TGA1_S02_16357	3.2	yes	0	1.70	agguaacgucugguggcaa
TGA1_S05_35063	2.9	yes	0	4.98	gcaagaucaaaacucaaa
TGA1_S17_175238	2.8	yes	4.34	13.62	uggaaguugaaucgagaagccc
TGA1_S09_88615	2.7	yes	0	1.70	cguguaccagagcgucau
TGA1_S08_81282	2.5	yes	0.60	0	uccgauagggauguucgggcu
TGA1_S20_238862	2.5	yes	4634.89	4921.04	uaggacuuuaccgugacgucc
TGA1_S22_257134	2.5	yes	1.45	12.20	cgguggauugaacgggacucuuggu
TGA1_S05_43489	2.4	yes	28.74	491.72	cucgggagaaggcggccu
TGA1_S07_78907	2.3	yes	0.36	0	gucuucuugaucucuuau
TGA1_S18_185930	2.3	yes	25.59	160.54	acccggcuuggagaugug
TGA1_S20_209761	2.3	yes	0.38	0	ugaugauucggcuaguucggacag
TGA1_S22_248446	2.3	yes	0.94	10.71	cggcgaggcuguguuucagcga
TGA1_S20_223477	2.2	yes	445.56	655.43	uacguaggacucuaccgugacau
TGA1_S20_238808	2.2	yes	44.36	38.21	guucgaggguugaaauga
TGA1_S06_55136	2	yes	0	1.70	auguaugccuccugagauua
TGA1_S16_151294	2	yes	0	1.70	acuucauccauagauaucgcaa
TGA1_S17_162471	2	yes	3.95	13.62	uggaaguugaaucgagaagccc
TGA1_S19_199524	1.9	yes	0.18	0	uaccucucauucacugcagug
TGA1_S02_9056	1.8	yes	1.77	26.56	ggaaacaagguuguucugacuau
TGA1_S13_112804	1.8	yes	0.60	0	uuaugugguacggcagagagu
TGA1_S04_31828	1.7	yes	0.30	23.35	gguucgacucccggcuugu
TGA1_S14_132761	1.6	yes	7.96	6.95	aacgugcaauugcuaccaa
TGA1_S20_229688	1.6	yes	2.62	5.62	ugagggaccggauucgcca
TGA1_S22_253299	1.6	yes	15.54	38.89	auccaaaagcucggcuuu
TGA1_S23_271212	1.5	yes	0	5.62	agcgaggacauuuaugac
TGA1_S05_33550	1.4	yes	2.47	27.03	accuagagaacgaugguucccauu
TGA1_S07_67387	1.3	yes	0.71	0	aagcagauugcgaggggucauuug
TGA1_S22_265703	1.1	yes	0.38	9.86	gggcagucuguuggacuccggu
TGA1_S07_76476	1	yes	1.14	0.85	cucggucuguuguggauugguc
TGA1_S19_189543	0.7	yes	1.14	0	aaggacauuuuggaggag
TGA1_S07_65040	0.1	yes	1.50	5.73	ucgaaguugugguuguagugguagu

## Data Availability

The data that support the findings of this study areavailable on request from the corresponding author.

## Supplementary Materials

The following are available online at https://www.mdpi.com/article/10.3390/jof7120997/s1, Figure S1: Knockout or over-expression of *Tr*-milRNA or gene in NJAU 4742. (a) Schematic diagram for *Tr*-milRNA or gene disruption by double crossover recombination; (b) schematic diagram for *Tr*-milRNA over-expression; (c) schematic diagram for gene over-expression; (d) analysis of expression level of *Tr*-milRNA relative to 18S in wt, OE-*Tr*-milRNA1, OE-Mut-*Tr*-milRNA1, and empty vector transformant (EV) strains determined by qPCR. (e) analysis of expression level of *Trvip36* gene relative to *Tef* in wt and OE-*Trvip36* strain determined by qPCR. Data were calculated from three biological replicates. Error bars represent ± SDs. * *p* < 0.05, ** *p* < 0.01, *** *p* < 0.001. A *P*-value < 0.05 was regarded as statistically significant. The expression values are normalized to wt; (f,g) verification of Δ*Tr*-milRNA1 (f), Δ*Trvip36* (g), wt-*egl*-GFP, Δ*Trvip36*-*egl*-GFP, wt-*cbh*-GFP, and Δ*Trvip36*-*cbh*-GFP (h) mutants by PCR to verify homologous recombination. Figure S2: Diagram of construction principle for prepaing the mutants of lignocellulases-eGFP fusion strains. Arm1 and Arm2 were used as two arms of homologous recombination and *Hygb* gene was used as the biomaker for screening. Figure S3: GO (a) and KEGG (b) enrichment of candidate *Tr*-milRNAs target genes. Figure S4: The average intensity of EGL-GFP and CBH-GFP fluorescence in the ER and Golgi apparatus of wt and Δ*Trvip36* after staining hyphae with ER-Tracker™ and BODIPY^TM^ TR Ceramide, as markers of ER and Golgi compartments. Data were calculated from all valid pixels in the ER and Golgi apparatus, and error bars represent ± SEs. * *p* < 0.05, ** *p* < 0.01, *** *p* < 0.001. A *p* value < 0.05 was regarded as statistically significant and ns refers to no significance. Table S1: All PCR primers used in this study.
