# Supplementary material for: Tr-milRNA1 Contributes to Lignocellulase Secretion under Heat Stress by Regulating the Lectin-Type Cargo Receptor Gene Trvip36 in Trichoderma guizhouence NJAU 4742
Source: J Fungi (Basel). 2021 Nov 23;7(12):997. doi: 10.3390/jof7120997 (PMC8704016; doi:10.3390/jof7120997)
Supplement: Supplementary file 1 [file jof-07-00997-s001.zip › jof-1432390-supplementary.pdf]

Supplementary Materials

Figure S1. Knockout or over-expression of *Tr*-milRNAs and genes in NJAU 4742. (a) Schematic diagram for *Tr*-milRNAs or genes disruption through double crossover recombination; (b) Schematic diagram for the over-expression of *Tr*-milRNAs; (c) Schematic diagram for gene over-expression; (d) Analysis of the expression level of *Tr*-milRNA relative to 18S in wt and OE-*Tr*-milRNA1 strains determined by qPCR. (e) Analysis of expression level of *Trvip36* gene relative to *Tef* in wt and OE-*Trvip36* strain determined by qPCR. Data were calculated from three biological replicates. Error bars represent ± SDs. *P<0.05, **P< 0.01, ***P<0.001. P-value < 0.05 is regarded as statistically significant. The expression values are normalized to wt; (f-g) Verification of Δ*Tr*-milRNA1 (f), Δ*Trvip36* (g), wt-*egl*-GFP, Δ*Trvip36*-*egl*-GFP, wt-*cbh*-GFP and Δ*Trvip36*-*cbh*-GFP (h) mutants by PCR to verify homologous recombination.

Figure S2. Diagram of construction principle for prepaing the mutants of lignocellulases-eGFP fusion strains. Arm1 and Arm2 were used as two arms of homologous recombination and *Hygb* gene was used as the biomaker for screening.

Figure S3. GO (a) and KEGG (b) enrichment of candidate *Tr*-milRNAs target genes.

Figure S4. The average intensity of EGL-GFP and CBH-GFP fluorescence in the ER and Golgi apparatus of wt and Δ*Trvip36* after staining hyphae with ER-Tracker™ and BODIPY™ TR Ceramide, as markers of ER and Golgi compartments. Data were calculated from all valid pixels in the ER and Golgi apparatus, and error bars represent ± SEs. *P < 0.05, **P < 0.01, ***P < 0.001. A P value < 0.05 was regarded as statistically significant and ns refers to no significance.

Table S1. All PCR primers used in this study.

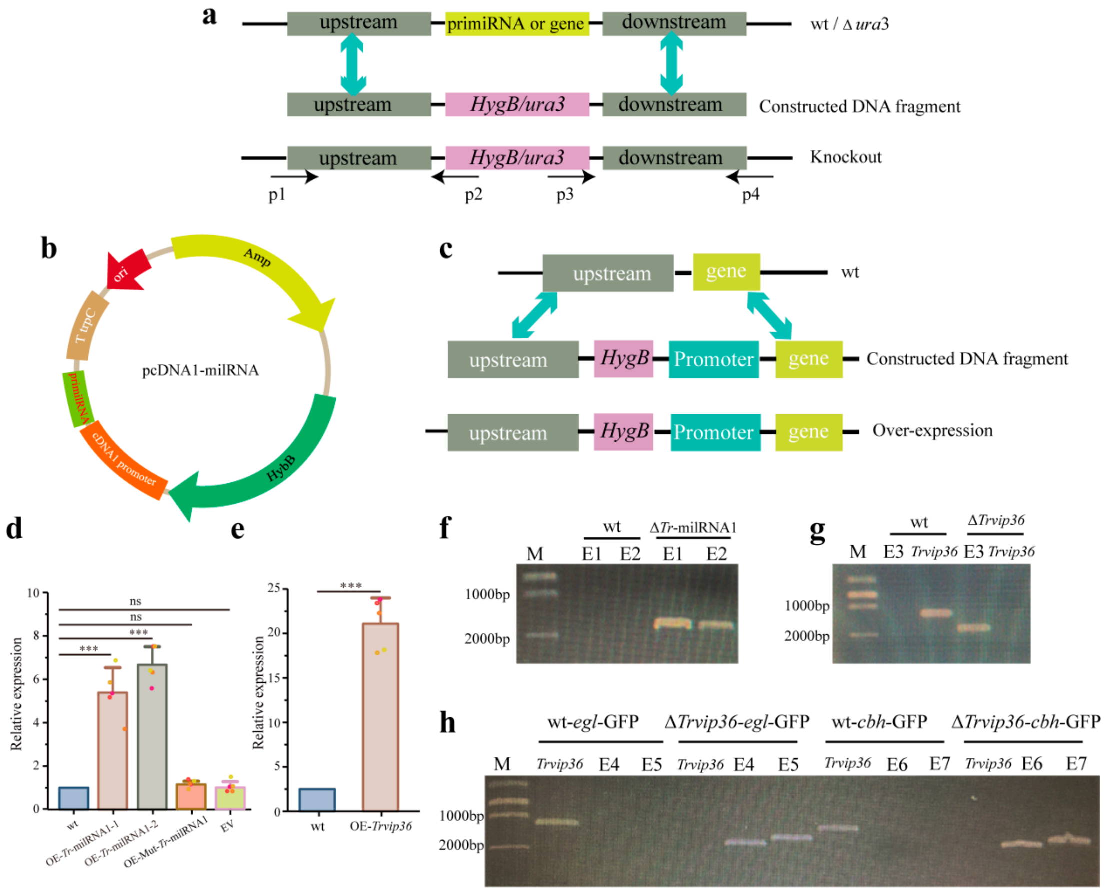

Figure S1. Knockout or over-expression of Tr-milRNAs and genes in NJAU 4742. (a) Schematic diagram for Tr-milRNAs or genes disruption through double crossover recombination; (b) Schematic diagram for the over-expression of Tr-milRNAs; (c) Schematic diagram for gene over-expression; (d) Analysis of the expression level of Tr-milRNA relative to 18S in wt and OE-Tr-milRNA1 strains determined by qPCR. (e) Analysis of expression level of Trvip36 gene relative to Tef in wt and OE-Trvip36 strain determined by qPCR. Data were calculated from three biological replicates. Error bars represent ± SDs. *P<0.05, **P< 0.01, ***P<0.001. P-value < 0.05 is regarded as statistically significant. The expression values are normalized to wt; (f-g) Verification of ΔTr-milRNA1 (f), ΔTrvip36 (g), wt-egl-GFP, ΔTrvip36-egl-GFP, wt-cbh-GFP and ΔTrvip36-cbh-GFP (h) mutants by PCR to verify homologous recombination.

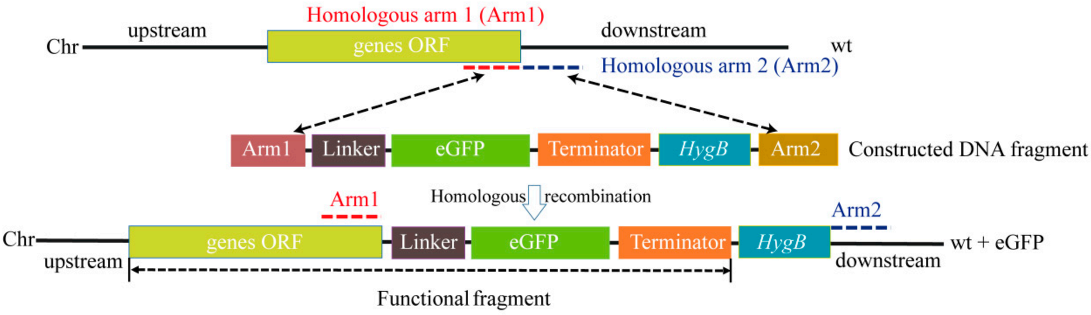

Figure S2. Diagram of construction principle for prepaing the mutants of lignocellulases-eGFP fusion strains. Arm1 and Arm2 were used as two arms of homologous recombination and Hygb gene was used as the biomaker for screening.

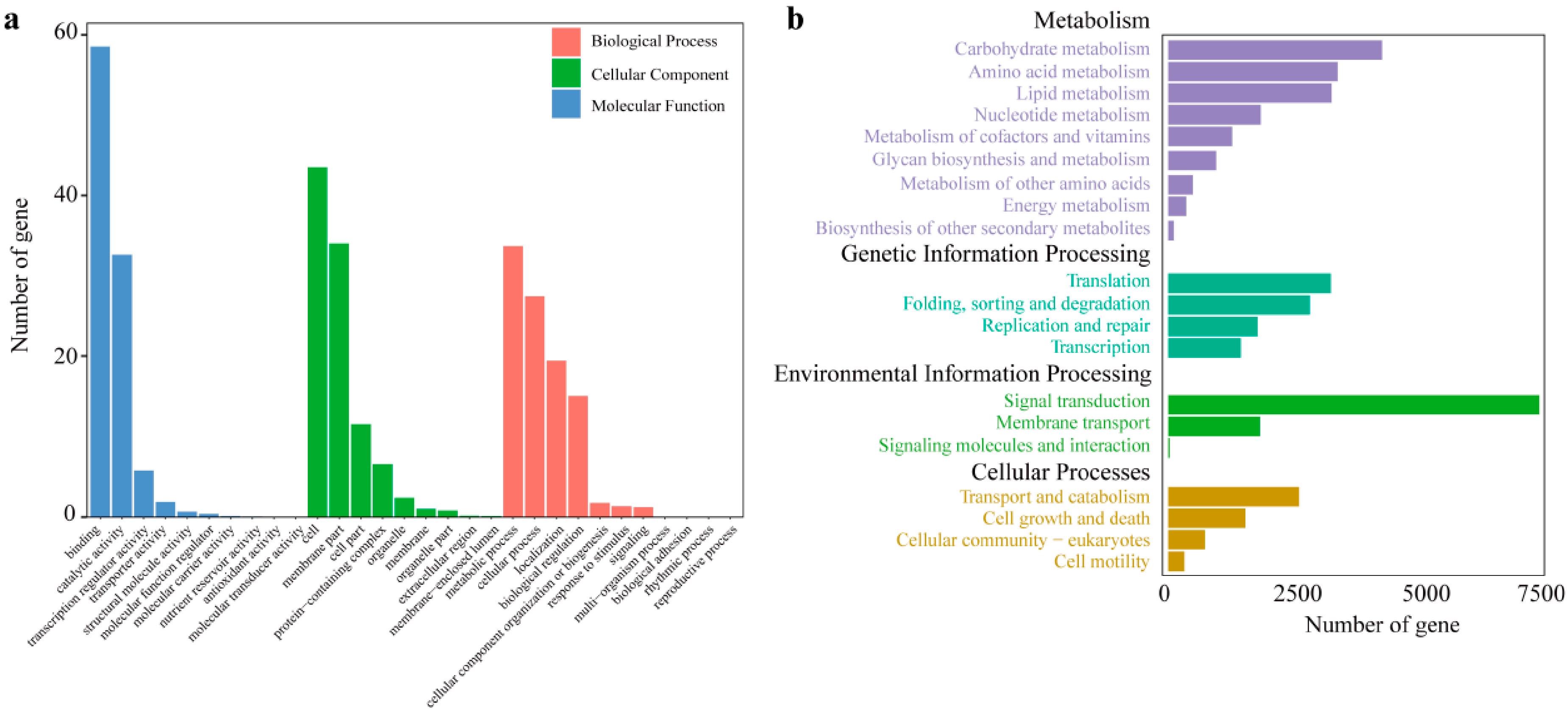

Figure S3. GO (a) and KEGG (b) enrichment of candidate Tr-milRNAs target genes.

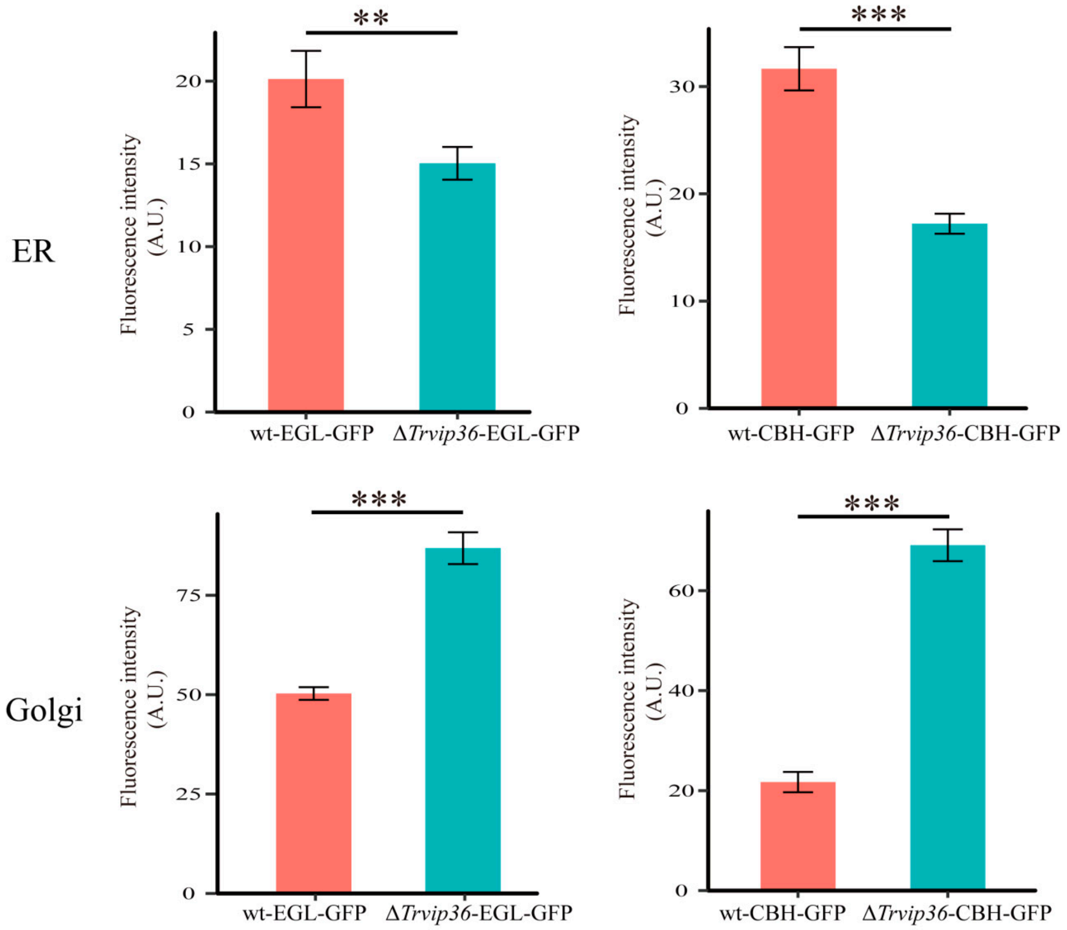

Figure S4. The average intensity of EGL-GFP and CBH-GFP fluorescence in the ER and Golgi apparatus of wt and ΔTrvip36 after staining hyphae with ER-Tracker™ and BODIPYTM TR Ceramide, as markers of ER and Golgi compartments. Data were calculated from all valid pixels in the ER and Golgi apparatus, and error bars represent ± SEs. *P < 0.05, **P < 0.01, ***P < 0.001. A P value < 0.05 was regarded as statistically significant and ns refers to no significance.

Table S1. All PCR primers used in this study.

**Primers**	**Sequence (5' - 3')**
**Primers for Δ*Tr*-milRNA1 construction and verification**	
E-*Tr*-miRNA1 (p1)	TGGTCGAGGCGAGAGGTCTC
E- *HygB*-R (p2)	CATCCTATCCAGATGGACATTGGAA
E- *HygB*-F (p3)	TCGTCCGAGGGCAAAGGAATAAT
E-*Tr*-miRNA1 (p4)	ATTGAGTGAGGAGTGACACTCTGG
HygB-F	GAGAGCTACCTTACATCAATATGGC
HygB-R	GGTACTATGGCTTAGATGGAATACCC
Tr-miRNA1-UF	TCTGCCAAGTGGGCAGTTGG
Tr-miRNA1-UR	GCCATATTGATGTAAGGTAGCTCTCTGCGGAGATTATTCAGCGCG
Tr-miRNA1-DF	GGGTATTCCATCTAAGCCATAGTACACGATATCATGGTGAAACGAGGCC
Tr-miRNA1-DR	CCACCACGTTCCGAACAAAGTAT
**Primers for OE-*Tr*-milRNA1 construction and verification**	
Tr-miRNA1-GF	CTCAAACTCCCAAAACAACCCACATACATCTGCTAGCTGCCATCCG
Tr-miRNA1-GR	TCCATCATTACACCCAGTCATCCCACTGCTGGGCAAGCCGTTTATG
Tr-miRNA1-G-DF	TGGGATGACTGGGTGTAATGATGGA
Tr-miRNA1-G-DR	ACCTAACAAACTTCACAGCTCCG
Promoter- F	GGGTATTCCATCTAAGCCATAGTACGAGAGAAGATGGCAGTGTAGAAGGG
Promoter- R	ATGTGGGTTGTTTTGGGAGTTTGAG
Tr-miRNA1-RT	GTCGTATCCAGTGCAGGGTCCGAGGTATTCGCACTGGATACGACACAAGC
Tr-miRNA1-RTPCR-F	GCGGGTTCGACTCCCG
Tr-miRNA1-RTPCR-R	AGTGCAGGGTCCGAGGTATT
Tef-F	TACAAGATCGGTGGTATTGGAAC
Tef-R	AGCTGCTCGTGGTGCATCTC
**Primers for Δ*Trvip36* and OE-*Trvip36* construction and verification**	
E-*Trvip36* (p1)	GGTTCGCTTCCGCGAGGA
E-*ura3*-R (p2)	CATCCAATGCAATGCATGCGAG
ura3-F	CAGTATGGTCAACTACGGTCCAGC

ura3-R	CGTATCTGATCAAGGAACGTTAGCG
Trvip36-F	ATGCGGCTCTCCTCGCTG
Trvip36-R	CTAGAATCTGTGGCTCTTGGTTCT
Trvip36-UF	ACAGGACATGGGACGAATCAGTAT
Trvip36-UR	GCTGGACCGTAGTTGACCATACTGGGTCACTTGGAGATCGGCACG
Trvip36-DF	CGCTAACGTTCCTTGATCAGATACGGGGAGATGTTGGACTCATCCAG
Trvip36-DR	GGTTTCCTTATCGCCCGGC
Promoter-F	GGACTGGGGTATTCCATCTAAGCCGAGAGAAGATGGCAGTGTAGAAGGG
Promoter-R	CAGCGAGGAGAGCCGCATATGTGGGTTGTTTTGGGAGTTTGAG
Trvip36 -RTPCR-F	ATCCACAAAGTCCTGCCTAA
Trvip36 -RTPCR-R	CGAACCTGAATACTGTCCCT
18S-F	AGACAAGGCGGGCAGCAAGA
18S-R	GCCATCAGGGTCAGCACAGAA
**Primers for the construction and verification of fluorescent strains**	
E-GFP	TGGTGCAGATGAACTTCAGGGTC
E-*egl*	ATGGTGCCCTTAACTGGATGGTAA
egl-F	AGAACAGGGATACCGACATCTCCA
egl-R	GCAGTATTGACATGCCGTTGG
egl-d-F	ACGAAGCCCATAGAGTAAGAAGTT
egl-d-R	ACATCGAGAATGACAGCGGC
E-*cbh*	CCAGGAGTTCACACTCTCTGGC
cbh-F	GAAGTTCATCAACGGTCAGGCC
cbh-R	CAGGCACTGAGAGTAGAATGGGTT
cbh-d-F	TTCTGATGCTAGCAAAGACGGTCAT
cbh-d-R	ACAGAGAACCCGGAACTGCA
